# Unlocking Engagement: Enhancing Participation in Research With Vulnerable Populations

**DOI:** 10.3389/ijph.2024.1606705

**Published:** 2024-10-31

**Authors:** Laura Rojas-Rozo, Geneviève Arsenault-Lapierre, Diane Dumaresq, Thérèse Trépanier, Paul Lea, Karen Myers Barnett, Denis O’Connor, Rosette Fernandez Loughlin, Kori Miskucza, Mary Beth Wighton, Claire Godard-Sebillotte, Andrea Gruneir, Jean-Baptiste Beuscart, Susan E. Bronskill, Nadia Sourial, Eric E. Smith, Jennifer Bethell, Isabelle Vedel, The COVID ROSA Research Team, Juanita Bacsu

**Affiliations:** ^1^ Department of Family Medicine, Faculty of Medicine and Health Sciences, McGill University, Montreal, Canada; ^2^ Lady Davis Institute for Medical Research, Jewish General Hospital, Montréal, QC, Canada; ^3^ Center for Research and Expertise in Social Gerontology, CIUSSS Centre-Ouest de l’Ile-de-Montréal, Montréal, QC, Canada; ^4^ Department of Medicine Division of Geriatrics, McGill University Health Center, Montreal, QC, Canada; ^5^ Faculty of Medicine and Dentistry, University of Alberta, Edmonton, AB, Canada; ^6^ CHU Lille, ULR 2694 - METRICS: Evaluation des Technologies de Santé et des Pratiques Médicales, Université de Lille, Lille, France; ^7^ Institute for Clinical Evaluative Sciences, Dalla Lana School of Public Health, University of Toronto, Toronto, ON, Canada; ^8^ Department of Health Management, Evaluation and Policy, School of Public Health, University of Montreal, Montreal, QC, Canada; ^9^ Cumming School of Medicine, University of Calgary, Calgary, AB, Canada; ^10^ KITE Research Institute, Toronto Rehabilitation Institute, University Health Network, Toronto, ON, Canada

**Keywords:** guide, dementia, vulnerability, patient engagement, participatory research

## Background

Traditionally, people directly affected by health conditions have often been relegated to the passive role of “subjects” in health research [1]. Conversely, participatory research is conducted with and for people with lived experiences (PWLE) [2], such as patients, families, or caregivers. PWLEs have invaluable knowledge about the condition under study, having encountered it personally [3]. Participatory research not only advances patient-centered research [4] but also improves the entire research process by making it more relevant, adequate, meaningful, and impactful for the researchers and the communities, while promoting inclusivity, relevance, and empowerment of the PWLE involved [5, 6].

It is well known that, while vulnerable and disadvantaged populations are more likely to experience poor health, they are less likely to be involved in public health research because of their social or physical location, health status, or the circumstances that make them more vulnerable [7–9]. However, involving such vulnerable populations ensures that research results do not favor the point of view of more advantaged groups; support the generation of study results that are more adequate, relevant, and empowering for vulnerable populations and the community [8]; enhance the representation of vulnerable groups, increase the visibility of their needs, and enable advocacy efforts on their behalf; and streamline the dissemination of research findings [8]. Furthermore, the engagement of vulnerable populations has been beneficial for the populations themselves, fostering a sense of empowerment as they are encouraged to voice their experiences and push for enhancements in their living conditions [10]. While guidance exists on how to engage specific vulnerable populations, such as people with low socio-economic status, victims of sex abuse, or asylum seekers [7, 10, 11], little is known about how to specifically engage people who are vulnerable due to health conditions.

## Objective

The objective of this paper is to describe a tailored method for the engagement of vulnerable people in participatory health research.

## Context

Our engagement activities were part of a project investigating the impact of the COVID-19 pandemic on health service use in people with dementia in Canada [12]. People with dementia face multiple barriers to their engagement in research due to several reasons, such as their cognitive impairments, internalized stigma and misconceptions, lack of opportunity and awareness of research opportunities [4, 13–15], physical limitations, increased dependence on their care partners [15], and attitudinal biases of researchers [16].

## Overview of Our Approach

By sharing our approach, we suggest that it is indeed possible to engage with such vulnerable populations using tailored strategies: 1) recruitment and status of PLWE in the research project, 2) involving PWLE throughout all phases of the research project, 3) designating a single research contact person for all communications with PWLE, 4) developing an appropriate onboarding strategy, 5) offering flexible engagement, and 6) adapting how meetings are conducted. The sequence in which these strategies are presented does not align with their respective priority and significance. Research teams are advised to assess both the significance and sequence of the following strategies based on their team’s specific needs and characteristics.

### Strategy 1: Recruitment and Status of PLWE in the Research Project

We recruited 16 PWLE (three of whom had mild-to-moderate dementia and 13 of whom were care partners) from multiple sources, including previous partnerships, through advertisement via the Quebec Federation of Alzheimer Societies, and through the Engagement of People with Lived Experience of Dementia program of the Canadian Consortium on Neurodegeneration in Aging. These 16 people were considered full research team members as co-researchers and, as such, a remuneration based on meeting participation was offered to all. Some refused this remuneration as it may have affected their pensions.

### Strategy 2: Involving PWLE During All Phases of the Research Project

**FIGURE 1:**
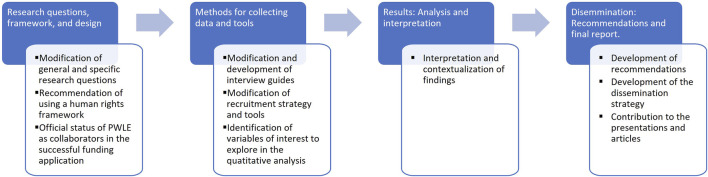
Research steps and impacts of the involvement of people with lived experience (Canada, 2024).

As recommended in participatory research [13, 14], PLWE were involved throughout the entire research process (see Figure 1). Our group was comprised of 42 co-researchers, including 16 PWLE, 16 academic researchers from nine universities, three students, and seven collaborators from various institutions such as supportive organizations and advocacy groups, public health agencies, provincial health governing bodies, and family physicians’ groups. Having a large group of PWLE ensured some level of consistency despite variability in participation and offered a bulwark against potential issues involving power dynamics observed in previous research [16].

All co-researchers, PWLE in particular, were invited to all steps of the project (design/research questions and framework, methods, results, and dissemination) [6]. Multiple meetings were held on regular occasions. We structured our project into a large executive committee, whereby general directions and interpretation of results were decided. PWLE were members of this committee to ensure their ownership of the whole research process [6]. Additionally, we organized distinct working groups, one per research objective, to discuss the data collection tools, analysis plans, and interpretation of results as they pertained to each specific objective. PWLE co-researchers were invited to each committee and working group. Large executive committee meetings, where every co-researcher was invited, were organized annually. Then, working groups were held, approximately bi-monthly, in which data collection, participant recruitment, and an analyses plan were discussed. These meetings involved any interested co-researcher (PWLE, researchers, students, and collaborators).

### Strategy 3: Designating a Single Contact Person for All Communications With PLWE

Good communication with PWLE is key [13, 14]. In our project, we tailored our communication channels and designated a single contact person with previous experience in participatory research to direct suggestions, questions, or comments to and from PWLE. This contact person was trained with the existing best practices for participatory research [13, 14]. This person coordinated with the PWLE to explain the consent form in detail and answer any PWLE question [17], arrange convenient dates, and circulate documents that adhered to Alzheimer Society of Canada’s patient-centered language recommendations [18]. They avoided acronyms, technical jargon, and complex terminology. Following each meeting, the contact person gathered additional comments from the PWLE that sometimes arose after the meeting and relayed these to the rest of the team. Furthermore, this person provided PWLE with regular updates and summaries to maintain a high level of involvement, either through emails or ad-hoc meetings. This deliberate approach facilitated a better comprehension of the discussed topics and actively fostered the participation of PWLE during meetings.

### Strategy 4: Developing an Appropriate Onboarding Strategy

As it is essential for all researchers and PWLE to be on the same page on the project [13, 14], we organized initial individual meetings between the contact person, PWLE, and two research coordinators to discuss our roles and our mutual expectations in terms of the extent and manners of involvement. We discussed explicit details regarding authorship and financial compensation to ensure transparency and fair acknowledgement of everyone’s time and contributions.

### Strategy 5: Offering Flexible Engagement

It is key to remain flexible with the level of involvement of PWLE [13]. To ensure flexibility and respect individual preferences, everyone was offered the opportunity to participate or not to the steering committee and to choose the working groups they wished to engage in. To adapt to the progressive nature of the disease and the variable availability and capacity of PWLE, everyone determined the amount of time they could allocate to the project.

### Strategy 6: Adapting How Meetings Were Conducted

It is important for PWLE to have a voice within the research team [14]. The first half of each steering committee meeting was dedicated to the input and questions of PWLE. The discussion was then open to all attendees: PWLE, researchers, students, and collaborators. Additionally, we ensured to send the necessary documentation at least 1 week in advance of each meeting, allowing everyone ample time to prepare.

## Impact of These Engagement Strategies

Our tailored approach was well-received by all: PWLE, researchers, students, and collaborators. We were able to take into account PWLE’s experiences to inform the research project in an ongoing and inclusive manner. Furthermore, the contribution of the PWLE to the project resulted in many impactful outcomes. For instance, we revised the research questions and framed the project in a human rights framework; we added equity-based stratifications to our quantitative analysis plan; and we built the interview guides with direct input from the PWLE (See Figure 1: Research steps and impacts of the involvement of persons with lived experience). PWLE and researchers, positively satisfied with the process, agreed to share their experience with the media, and many are co-authors on this paper.

## Conclusion

Our experience revealed that the potential issues related to engagement of PWLE in participatory research are mitigated through adequate planning, open dialogue, and the provision of necessary support. Furthermore, it was useful to recruit a large group of PWLE and dedicate meeting time to hear their voices to avoid power dynamics between PWLE and academic researchers. However, this type of approach is time consuming, necessitating the dedication of important resources, including a trained contact person, and efforts to develop and implement a meaningful engagement strategy and to reconcile various opinions.

Nevertheless, using this engagement strategy enhanced the research process; it became more inclusive and ultimately more fruitful for all parties involved. Our approach to engaging vulnerable PWLE in health research not only fostered meaningful contributions from PWLE but also prompted valuable adjustments and enhancements to our research project.

## Group Members of The COVID ROSA Research Team

Juanita Bacsu, Howard Bergman, Joanne Bracken, Matthieu Calafiore, Yves Couturier, Nouha Ben Gaied, Serge Gauthier, Mario Gregorio, Sid Feldman, Ngozi Iroanyah, Aaron Jones, Vladimir Khanassov, Julie Kosteniuk, Ginny Lane, Laura C. Maclagan, Carrie McAiney, Debra Morgan, Megan O’Connell, Manuel Montero Odasso, Lisa Poole, Jacqueline Quail, Pamela Roach, Dallas Seitz, Saskia Sivananthan, Natalia Smith, Machelle Wilchesky.
